# A review on morphotropic phase boundary in fluorite-structure hafnia towards DRAM technology

**DOI:** 10.1186/s40580-022-00333-7

**Published:** 2022-10-01

**Authors:** Minhyun Jung, Venkateswarlu Gaddam, Sanghun Jeon

**Affiliations:** School of Electrical Engineering, Korea Advanced Institute of Science & Technology, 34141 Daejeon, Republic of Korea

**Keywords:** Ferroelectric fluorite structures, Hafnia material, Morphotropic phase boundary, MFM capacitors, DRAM technology

## Abstract

**Abstract:**

In the present hyper-scaling era, memory technology is advancing owing to the demand for high-performance computing and storage devices. As a result, continuous work on conventional semiconductor-process-compatible ferroelectric memory devices such as ferroelectric field-effect transistors, ferroelectric random-access memory, and dynamic random-access memory (DRAM) cell capacitors is ongoing. To operate high-performance computing devices, high-density, high-speed, and reliable memory devices such as DRAMs are required. Consequently, considerable attention has been devoted to the enhanced high dielectric constant and reduced equivalent oxide thickness (EOT) of DRAM cell capacitors. The advancement of ferroelectric hafnia has enabled the development of various devices, such as ferroelectric memories, piezoelectric sensors, and energy harvesters. Therefore, in this review, we focus the morphotropic phase boundary (MPB) between ferroelectric orthorhombic and tetragonal phases, where we can achieve a high dielectric constant and thereby reduce the EOT. We also present the role of the MPB in perovskite and fluorite structures as well as the history of the MPB phase. We also address the different approaches for achieving the MPB phase in a hafnia material system. Subsequently, we review the critical issues in DRAM technology using hafnia materials. Finally, we present various applications of the hafnia material system near the MPB, such as memory, sensors, and energy harvesters.

**Graphical Abstract:**

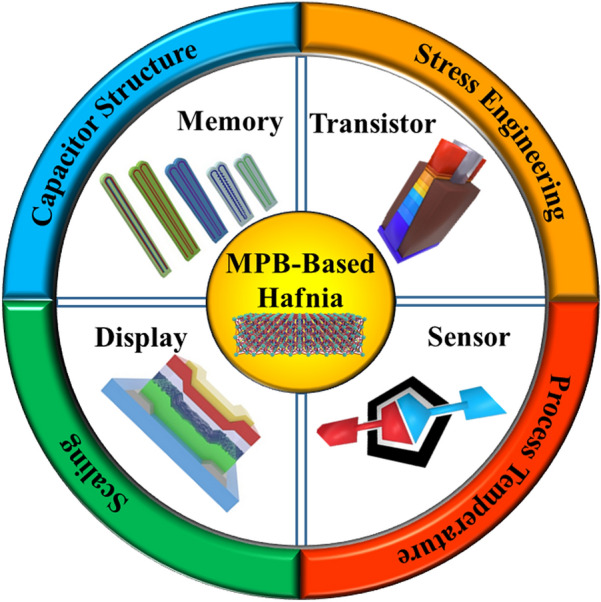

## Introduction

### Perovskite and fluorite structure ferroelectrics

A ferroelectric material can maintain its spontaneous polarization characteristics without an external electric field and can generate an electrical signal in response to pressure (piezoelectric effect) and heat (pyroelectric effect) [1, 2]. Owing to the remarkable properties of ferroelectrics, various materials and processes for industrial applications have been investigated over the past decades. The morphotropic phase boundary (MPB), which refers to the interface between two different crystal phases of a ferroelectrics polar and tetragonal nonpolar symmetries, has received considerable attention because of the abrupt increase in the dielectric and piezoelectric constants occurring in this region. The discovery of the MPB can be traced back to the 1950s, when PbZrO_3_-PbTiO_3_ (PZT), a representative ferroelectric with a perovskite (ABO_3_) structure, was developed. PZT has been extensively studied owing to its excellent ferroelectric properties in terms of composition and process. In particular, the MPB of PZT appears at the boundary between tetragonal and rhombohedral crystal phases [3, 4]. This intere{Singh, 2006 #1}sting change in electrical properties is due to the unstable lattice and mechanical properties (softening elastic modulus) caused by the polarization movement of the metastable state at the compositional interface [5, 6]. However, perovskite-based ferroelectrics present a few issues, such as the harmful elements (lead and cadmium) required to increase the Curie temperature, minimum film thickness required for ferroelectric behavior which limits various applications.

The MPB with a fluorite (AB_2_) structure can overcome the disadvantages of perovskite MPB. In particular, ZrO_2_ and HfO_2_ have attracted considerable attention in the last decade owing to their excellent compatibility with conventional complementary metal oxide semiconductor (CMOS) processes and the ease of deposition with atomic layer deposition (ALD) [7–10]. The origin of ferroelectricity in perovskite-structured materials is the mobile metal ion in the asymmetric molecular structure (c-axis tension in the crystal structure). However, the eccentricity of oxygen ions (a-axis tension) can induce ferroelectricity in the fluorite structure [11]. Despite the different origins of ferroelectricity, a phenomenon in the MPB similar to that of the perovskite material was found in the fluorite-structure MPB. This interesting property in the MBP region where the dielectric activity is maximal can be adopted as a cell capacitor in dynamic random-access memory (DRAM).

### MPB in fluorite-structure hafnia

The MPB is a mixed crystalline phase, which exists between the phases of ferroelectric (orthorhombic; o-phase) and anti-ferroelectric (tetragonal; t-phase) in the hafnia materials system. Consequently, the dielectric constant (κ) is maximized near the MPB, resulting in a reduced equivalent oxide thickness (EOT) [12]. The MPB has recently emerged as a novel strategy for achieving high κ and reducing the EOT in hafnia materials. One of the approaches to reduce EOT is the thinning of the high-κ layer. However, while thinning Hf-Zr-O (HZO) films leads to increase the capacitance/dielectric constant, it also results in increasing the leakage current density and thereby causes reliability issues [13]. Therefore, currently, pursuing high-κ dielectrics in various alternative ways is the key approach for increasing the dielectric constant and decreasing the EOT. Various approaches have been adopted for improving the dielectric constant of hafnia materials, including doping with various elements, such as Si, Zr, and Al; adjusting the fabrication process; manipulating the surface and interface; using field cycling effects; designing different capacitor stacks, such as nano-laminates and super-lattices; and introducing new materials [14–17]. Additionally, the dielectric constant was improved by optimizing various factors, such as the doping concentration, ozone dosage, strain, and temperature in the ferroelectric hafnia material system [18]. Park et al. [12] presented their work on HfO_2_ and ZrO_2_ compositions, which indicated how to obtain high κ and low EOT near the MPB [12]. Very recently, in 2021, Zhou et al. reported Al-doped HfO_2_ films with a record-high κ value (~ 68) near the MPB phase by deposition temperature engineering [19]. In this study, a very high thermal budget (750 °C, 30 s) was used to achieve a high κ value. Even though the dielectric constant was very high, the EOT was not adequate at the required specification of leakage current density.

### Recent advancements in DRAM technology

DRAM is a key element of semiconductor-based memory, and its performance has been improved over the past few decades. In addition, with the emergence of the 4th industrial revolution, the market demand for technologies such as artificial intelligence, the Internet of Things (IoT), and cloud computing is increasing. To operate modern computers, DRAM must have high density (> 16 GB), high-speed operation (~ 20 ns), and excellent reliability characteristics, including an ultimate endurance of 10^16^ cycles [13]. To fulfill these demands, a novel breakthrough technology is required to overcome the limitations of device miniaturization [20]. While shrinking the dimensions of the DRAM cell capacitor, several factors must be considered, such as sufficient cell capacitance, low leakage current, and data retention time. Scaling the EOT while maintaining a low leakage current density (10^–7^ A/cm^2^ at 0.8 V) is one of the key requirements for DRAM technology [21]. High-κ materials such as TiO_2_ and SrTiO_3_ have been extensively studied to achieve a high-κ value of greater than 100 [22–25]. However, despite having high-κ value, these materials are not compatible with TiN electrodes and have a high leakage current density. Another approach is selecting appropriate electrodes or engineering the electrodes to reduce the leakage current density. Currently, the ALD-grown TiN electrode is the most popular electrode in DRAM technology. However, this electrode has an insufficient work function and is not helpful for the reduction of current density. Therefore, alternative electrodes, such as noble metal (Ru) and conducting oxide electrodes (RuO_2_ and SrRuO_3_), are in demand [26]. Currently, Al-doped ZrO_2_ capacitors (TiN/AZO/TiN or TiN/ZrO_2_/Al_2_O_3_/ZrO_2_/TiN) are commercially available for DRAM technology. This structure has been successful in most technology nodes. However, the main problem with this structure is that it is limited to an EOT in the range of ~ 6–7 Å. Therefore, alternative capacitor structures and high-κ materials are required for this purpose [13, 27]. In addition, a thin film with high step coverage and high-quality films at low temperatures can be realized using the ALD process for the manufacture of advanced capacitor cells with a three-dimensional pillar structure. Further, MPB with fluorite structure has become an interesting property because the dielectric constant increases as the thickness of the thin film decreases to a certain level. Currently, tailoring the dielectric property in the MPB has become a novel strategy to scale down the EOT in hafnia when compared with traditional high-κ materials.

Therefore, the present review emphasizes the MPB of fluorite-structure hafnia. Herein, the origin of the MPB between perovskite and fluorite structures is explained in terms of physical aspects. Additionally, the formation of the MPB in hafnia thin films can be explained by various factors, such as thin-film structure, composition, deposition temperature, and heat treatment. We also present the critical issues encountered while achieving a high-κ and low EOT in fluorite structures. Finally, we present various applications near the MPB, such as memory, sensors, and energy harvesters.

## History and road map of morphotropic phase boundary between ferroelectric polar and symmetric phases

The discovery of the MPB is traced back to the 1950s when PZT, a representative ferroelectric with a perovskite (ABO_3_) structure, was developed. PZT has been extensively studied owing to its excellent ferroelectric properties in terms of its composition and process. In particular, the composition ratio is the main factor affecting the formation of the MPB [28]. The concept and experimental analysis of the MPB were developed over the past few decades [29]. After another decade, a theoretical analysis of the MPB in PZT has been presented [30]. Iwatra et al. clarified the abruptly increased dielectric property in MPB using the Landau–Devonshire theory based on the free energy point of view. To further understand the MPB, the temperature and pressure ambient dependence on PbTiO_3_ was investigated [5, 31]. They also suggested that the chemical pressure originated from the substitution of atoms with different atomic radii in PbTiO_3_. Here, the chemical pressure is an internal force caused by lattice strain with doping. In the case of PbTiO_3_, the Pb represents A-site and Ti represents B-site. The role of A-site is well known that the stabilization of ferroelectricity with hybridization between the unoccupied A-site ion and the oxygen ion result in octahedral rotations. On the other hand, the polarizability through the B-site can affect the balance between rhombohedral and tetragonal ferroelectric ground states [32]. However, new demands are emerging as the material properties reach their limits. In addition to other perovskite ferroelectrics, Pb(Mg_1/3_Nb_2/3_)O_3_–PbTiO_3_ (PMN–PT) and BaTiO_3_ or wurtzite-structured materials such as ZnO, Al_2_O_3_, and AlN have been reported along with many other approaches [4]. However, the complexity of various composite materials, as well as the limitations of thin-film applications, have proved to be a bottleneck.

**Fig. 1:**
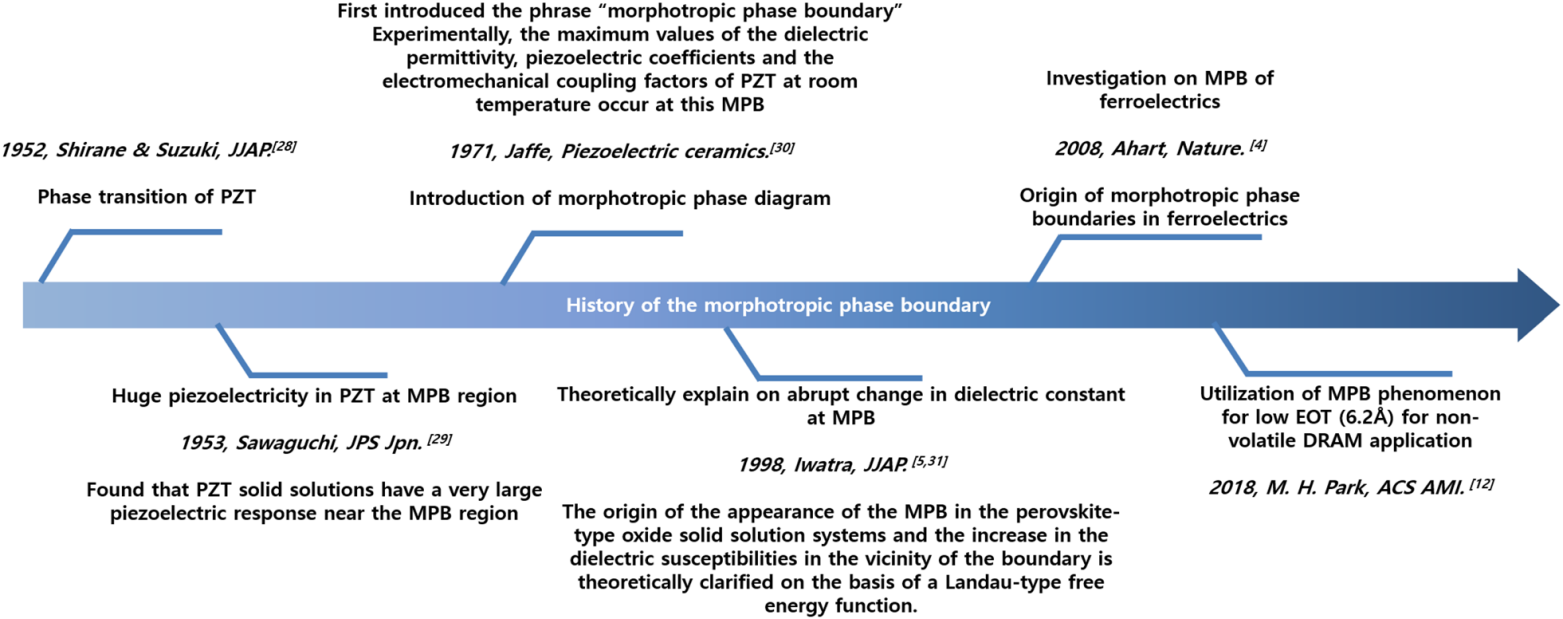
The road map of MPB history

Meanwhile, hafnia materials with fluorite structure have received significant attention because of their ferroelectricity at relatively thin thickness (~ 10 nm) [8, 10, 33–36]. In recent years, a substantial amount of research on hafnia has been reported because thin films with a fluorite structure have a high dielectric constant and semiconductor process compatibility in ultrathin film, which is required in the field of memory devices [18, 37–40]. In particular, an extremely low EOT of 6.2 Å was reported near the MPB in the HZO material system [12]. The road map of MPB history for both perovskite- and fluorite-structure ferroelectrics is shown in Fig. 1. Because HZO has a high coercive electric field and curie temperature than PZT, it can be utilized in a wider area than PZT. However, hafnia needs to be further investigated for adoption in commercial areas.

### Physical aspects of perovskite- and fluorite-structure MPB

**Fig. 2:**
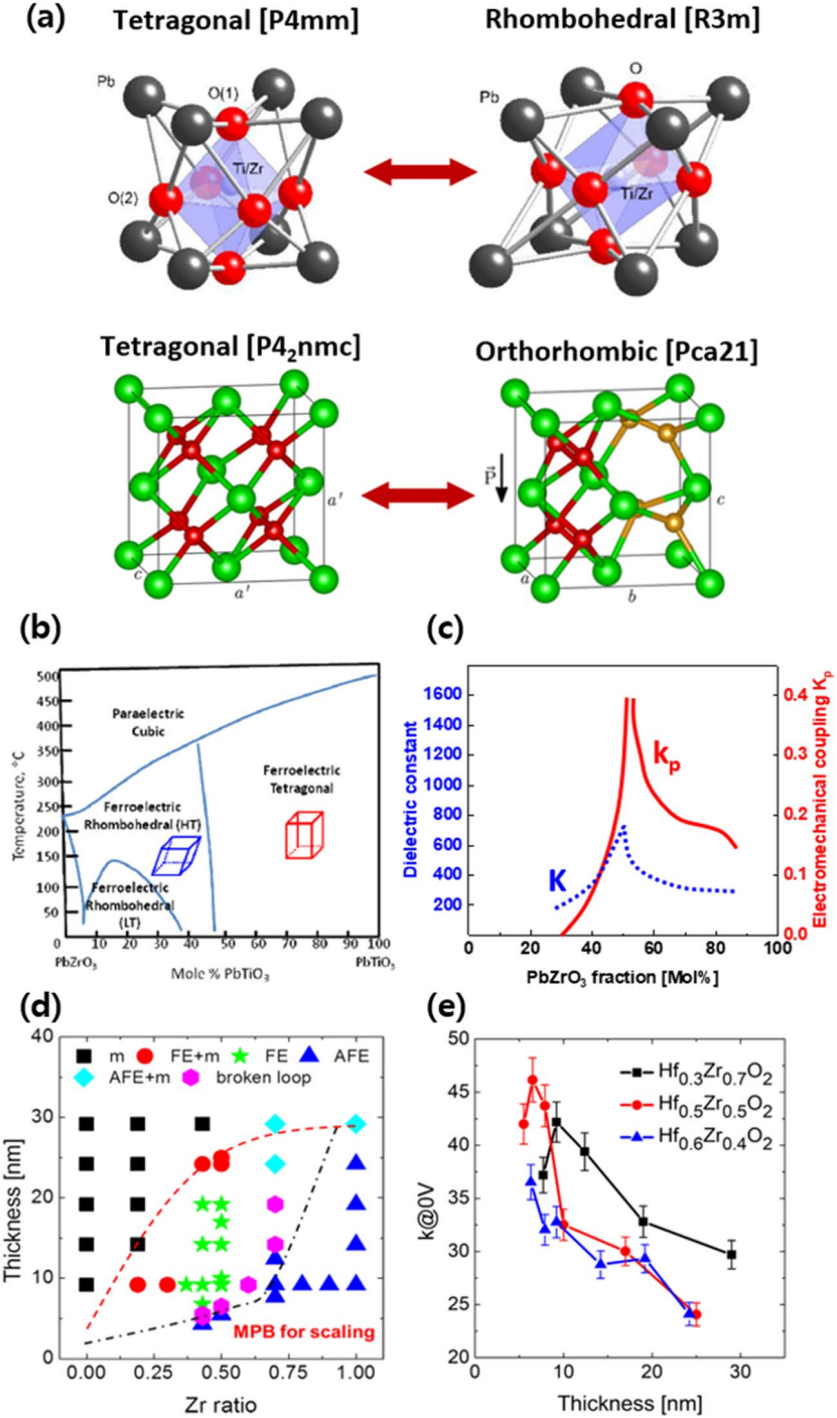
Difference between perovskite- and fluorite-structure ferroelectrics. **a** Two different crystalline phases at the interface of MPB with different atomic structure in PZT (PbZrO_3_ − PbTiO_3_, upper row), and HZO (lower row). **b** Phase diagram and **c** electro-mechanical properties of PZT with various PbZrO_3_ mol %. **d** Phase and **e** dielectric constant of HZO system with different thickness and Zr ratio. **a** was reproduced under a Creative Commons Attribution 3.0 Unported License [40] and AIP Publishing LLC, copyright 2015 [41] respectively. **b** was reprinted under a Creative Commons Attribution 3.0 Unported License [42] copyright 2015. **c**–**e** were reproduced with permission from [12]. Copyright 2018 American Chemical Society

As shown in Fig. 2a, the MPB of PZT appears at the boundary of the tetragonal and rhombohedral crystal phases, whereas in the hafnia thin film with fluorite structure, the MPB is observed at the boundary between the tetragonal and orthorhombic phases [41, 42]. At a relatively high thickness (~ 1 µm) close to the bulk material, the PZT maintains the MPB without being significantly affected by the temperature under T_c_. The phases of the material are mainly determined by the composition of the PZT (shown in Fig. 2b and c), whereas in the case of HZO, the phases are distributed not only by the composition but also by the film thickness and temperature of the thin film, as shown in Fig. 2d and e [12, 43]. Therefore, it is necessary to select an appropriate thin-film structure with a high dielectric constant in the hafnia fluorite structure.

**Fig. 3:**
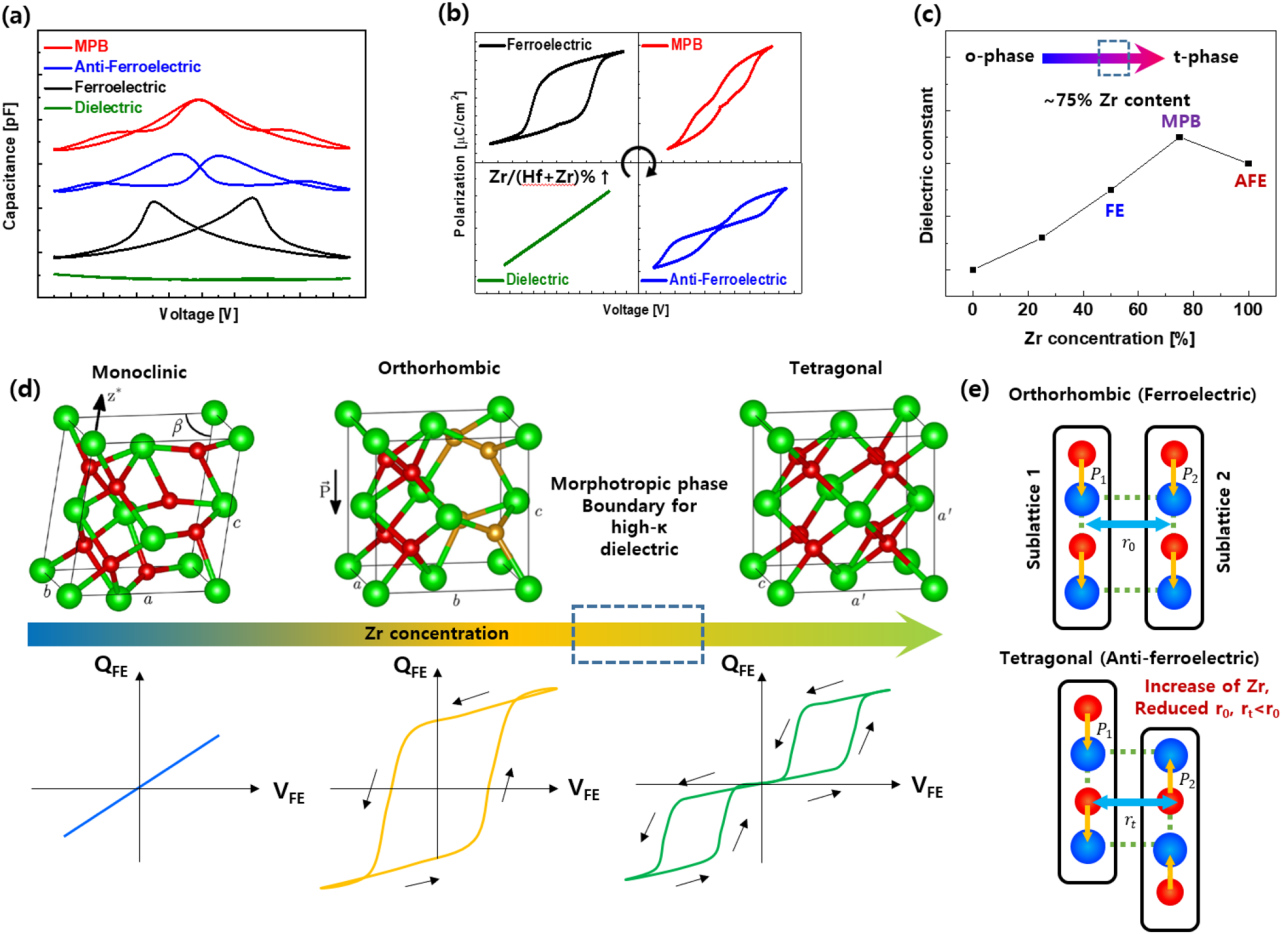
Electrical and materials characteristics of HZO thin film. **a** Capacitance and **b** polarization versus voltage curves of HZO thin film with various dielectric behavior at different composition of Zr. **c** Dielectric constant versus Zr concentration for HZO. **d** Electrical characteristics and **e** lattice interaction of each phase depending on Zr concentration at HZO system. **d** and **e** were redrawn from [43] and simulation data of atomic structure was reproduced with permission from [41]. Copyright 2015 AIP Publishing LLC

We carried out further investigations to confirm the formation of MPB in fluorite-structure hafnia. We analyzed various dielectric properties using the dielectric capacitance vs voltage (C–V) curve, as shown in Fig. 3a. In contrast to paraelectric materials, which are relatively less affected by electric fields, typical butterfly shaped curves and ribbon-shaped characteristics with two inflections are observed in ferroelectric and anti-ferroelectric materials. Interestingly, for the MPB, the highest single permittivity peak was achieved near 0 V. There is also a clear difference in the polarization–electric field (P-E) characteristics when evaluating the typical characteristics of ferroelectrics, as shown in Fig. 3b. In particular, we found the phase transition from dielectric to anti-ferroelectric in the HZO system as we increase the ratio of Zr to Hf. The MPB shows a pinched P-E curve, which is the intermediate shape between the ferroelectric and anti-ferroelectric properties. This can be explained by the neutral characteristics at the boundary between the o- and t-phases [18]. As a result, the extracted dielectric constant shows a maximum value at a high Zr ratio. (Fig. 3c).

In terms of crystallography, the higher the Hf concentration was, the more the monoclinic (m-phase; paraelectric) phase was promoted. We found that the formation of the o-phase is dominant as the portion of Hf and Zr becomes similar. In this structure, the oxygen ions bonded to the three metal atoms can be moved by the electric field, which is the origin of ferroelectricity. However, as the doping ratio of Zr in HZO is increased, the phase transition occurs from o-phase to tetragonal (t-phase; anti-ferroelectric) phase showing a double-polarization curve (shown in Fig. 3d) [44]. When an electric field above a certain level is applied to hafnia, the t-phase can be transformed to the o-phase. Consequently, an appropriate concentration of Zr in HZO leads to the formation of the MPB between the o- and t-phases. The increase in the ratio of Zr makes Hf and Zr-O bonds stretch. As shown in Fig. 3e, the polarization between sub-lattice sites is aligned in the same direction, resulting in ferroelectrics (sub-lattice spacing, r_0_). However, a relatively high Zr ratio shows a short sub-lattice spacing with compressive stress (r_t_ < r_0_). As a result, undesired increase in the dipole interaction energy caused by the bonds between two sub-lattices are compressed. Also result in phase transition from orthorhombic to tetragonal by switching the polarization in one sublattice. The reason of this phenomenon is related with larger lattice parameter of ZrO_2_ rather than HfO_2_. Moreover, it is evident that when Zr concentration rises (to 70%), the total energy decreases both diagonally and anti-diagonally. It shows the coexistence of both ferroelectric and anti-ferroelectric phases. The presence of MPB near this concentration was confirmed with the vanishing of energy barrier separating the two phases. In this case, the o-phase is transformed to the t-phase owing to the increased dipole interaction, while reducing the elastic energy. As a result, the total energy in a system can be expressed as the sum of the local energy (including electrostatic and Landau free energy) and the interaction energy (including dipole–dipole interaction and elastic energy) [42].

**Fig. 4:**
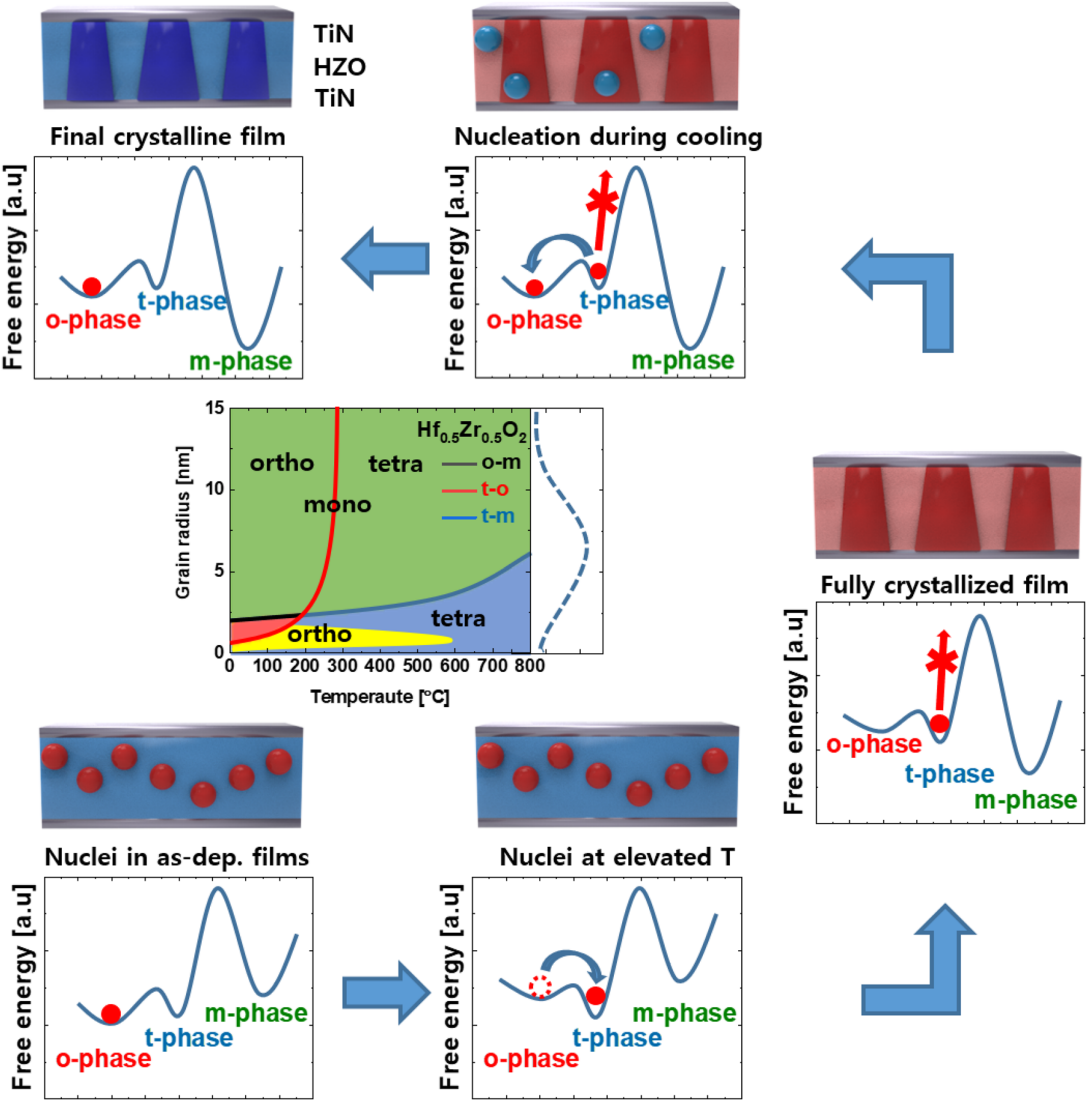
Phase transition of HZO during crystallization. Schematic diagram of crystallization of HZO thin film during rapid thermal annealing and cooling processes. This figure is redrawn from Ref. [44]

In order to obtain specific characteristics of HZO thin films, we need to consider various process factors such as thickness, composition, deposition temperature, and heat treatment. Figure 4 shows thermodynamically stable and metastable phases of HZO in grain size versus temperature. From a thermodynamic perspective, one can’t explain the phase transition and the stable phase of HZO. Instead, we need to consider a kinetic energy barrier to explain the phase transition of HZO observed in experiments [45]. After the deposition, the HZO is formed of a metastable phase with an amorphous structure. This is known to obey Oswartz’s law, in which thermodynamically metastable nucleation occurs preferentially over the most stable phase. Rapid thermal annealing (RTA) is a widely used method to form the metastable ferroelectric phase in hafnia. When the hafnia sample is thermally treated by RTA process, the t-phase is stabilized at high temperature. Below a certain temperature, for instance, 800 °C for HZO, the kinetic energy barrier from t-phase to m-phase is too huge, thereby suppressing the transition toward irreversible m-phase. After a sufficient period of time, the ferroelectric phase, which returns to the o-phase during the cooling process, forms a polycrystalline structure. As shown in the grain size–temperature diagram, rapid cooling and heat treatment can be considered to form the desired crystalline phase [45–48].

**Fig. 5:**
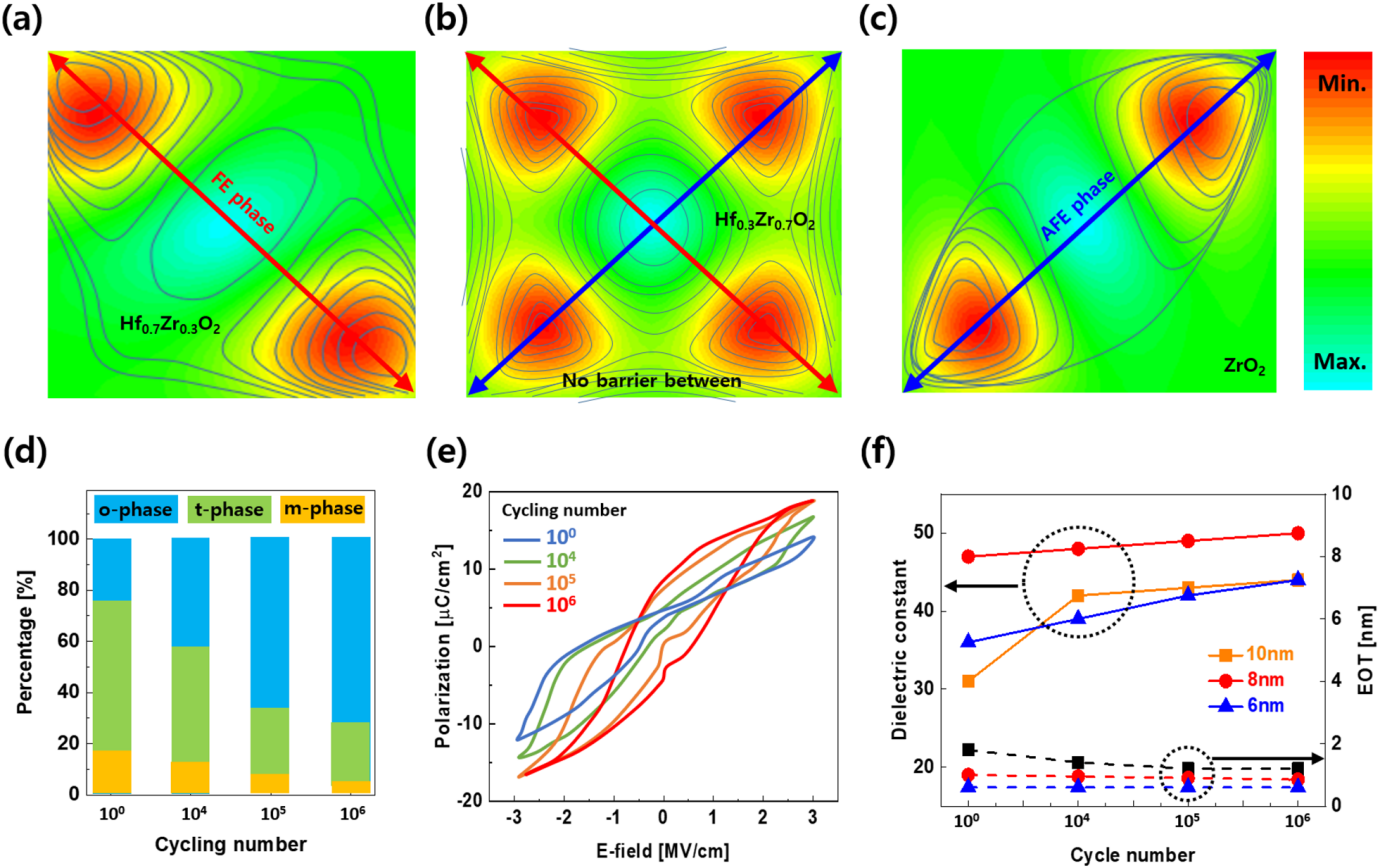
Phase transition in HZO. The total system energy contour of HZO system with different phase transformation **a** ferroelectric, **b** MPB with no barrier, and **c** anti-ferroelectric. **d** The evolution of phases of hafnia with cycling number. **e** Polarization versus electric field of hafnia with cycling number. **f** Dielectric constant and equivalent oxide thickness (EOT) for various thickness of hafnia with cycling number. **a**–**c** and **d**–**f** were reproduced from [17, 43] respectively

Next, we consider the electrical properties of MPB in more detail. To further understand the behavior of the dielectric in an actual device, we investigated its electrical behavior under an applied electric field. In some cases, ferroelectric capacitor requires wake-up process to align the net polarization in one direction after the fabrication procedure. According to previous report [49], the change in dielectric properties that occurs during this process is due to the redistribution of oxygen vacancy in the thin film. Depending on the ratio of Zr to Hf in the HZO thin film, the initial P–E curve shows dielectric, ferroelectric or anti-ferroelectric behavior. In all cases, the t-phase in hafnia plays very important role in the formation of MPB. After a repeated electrical pulse (wake-up), the device shows MPB characteristics with a single maximum capacitance on the C–V curve along with a pinched P–E curve. This is also known as field-induced phase transition. In addition, because the energy delivered by this electrical stimulation is not sufficiently large to overcome the kinetic energy barrier from t-phase to m-phase, it exhibits a reversible phase transition behavior from t-phase to o-phase (or vice versa) in the low electric field region. An analysis of the total energy contour of the HZO with the composition is shown in Fig. 5a–c. While the minimum energy lies in the anti-diagonal direction with ferroelectric characteristics in Hf_0.7_Zr_0.3_O_2_, it was found that there is no energy barrier between the ferroelectric and anti-ferroelectric in the MPB region (Hf_0.3_Zr_0.7_O_2_). Eventually, the minimum energy is on the diagonal with the anti-ferroelectric behavior in ZrO_2_. This transformation occurs because of compressive stress (increasing dipole interaction) with increasing Zr content.

In addition, Fig. 5d and e show the phase fraction and polarization curves of HZO with field cycling process, respectively. In contrast to the dominance of the t-phase in the pristine state, the o-phase increases under the influence of the phase transition with the electric field. This results in the increase in remanent polarization (P_r_). An investigation of different thicknesses was also performed. The increase in the dielectric constant and the consequent decrease in the EOT were confirmed as the electric field was repeated for all samples [44].

### Approaches to achieve MPB in fluorite structure hafnia

#### MPB in nano-laminates and super-lattices of hafnia

**Fig. 6:**
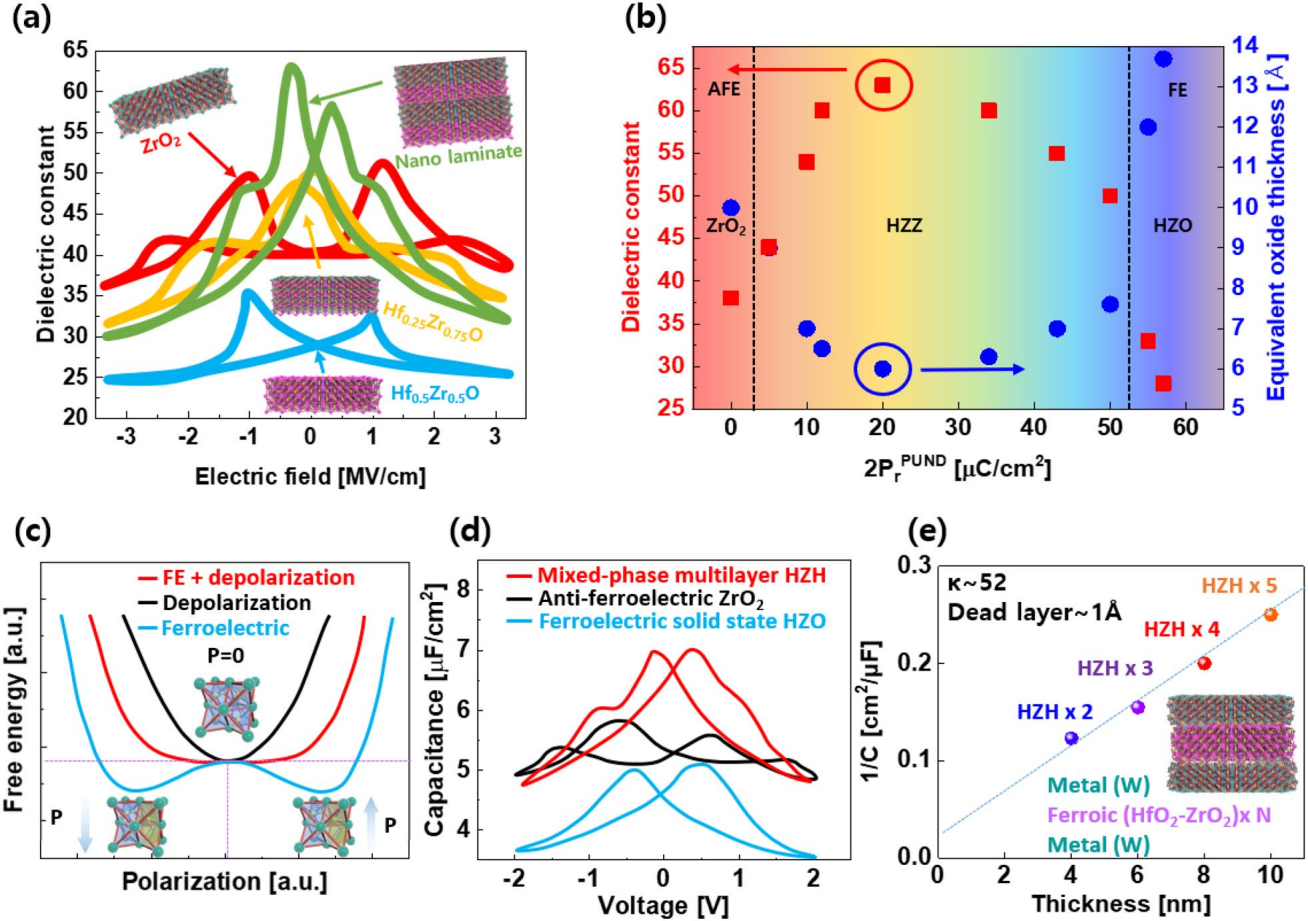
Structure dependence on HZO thin film. **a** ε_r_-electric field curves and schematics of HZO with different configuration (Green sphere: Zr, pink: Hf, blue: O). ε_r_ and EOT-2P_r_ PUND (positive up negative down), HZZ represents nano-laminate of HZO-ZrO_2_. **c** Energy landscape and **d** capacitance of different HfO_2_-ZrO_2_ structure. (e) Inversed capacitance value with repeated super lattice of HfO_2_-ZrO_2_ multi-layer. **a** and **b** were redrawn from Ref. [14, 15] respectively. **c**–**e** were redrawn from Ref. [16]

Conventional Al_2_O_3_, ZrO_2_, HfO_2_, and nano-laminate structures (ZAZ, HZH, and AZO) have been adopted to suppress the leakage current. However, the formation of the MPB in nano-laminate thin films between heterogeneous ferroelectrics has been reported [50, 51]. The lamination method for producing a thin film was developed to prevent crystal growth into undesired phases, which was adopted for the formation of MPB. Both the typical ferroelectric and anti-ferroelectric properties are present in the laminate structure of HZO-ZrO_2_, as shown in Fig. 6a, but it has a higher dielectric constant at zero electric field than a single-layer thin film. As shown in Fig. 6a, single-layer ferroelectric HZO shows a small dielectric constant (~ 27) when compared with that (~ 40) of the anti-ferroelectric (ZrO_2_). In contrast, the Zr-rich single-layer film (Hf_0.25_Zr_0.75_O_2_) shows a high dielectric constant (~ 50) when compared with ferroelectric (HZO) and anti-ferroelectric (ZrO_2_) devices, owing to the formation of MPB. When compared to other single-layer structures, HZO-ZrO_2_ nano-laminate structures all have a high dielectric constant (60), which is caused by the creation of the MPB (mixed o/t phase), which contains a significant amount of the t-phase [14, 15]. These properties were observed through quantitative examination of the retrieved EOT and dielectric constant values in the region where the P_r_ value was relatively low. This indicates a correlation with P_r_, which decreases at 0 V in the polarization curve. The MPB displays a characteristic resembling that of a ferroelectric material as the P_r_ value rises. On the other hand, as the P_r_ value approaches 0, paraelectric behavior caused by the t-phase in anti-ferroelectric is proven (Fig. 6b). In contrast to their typical short lifespan (~ 10^11^ cycles), fluorite-structured ferroelectrics must meet the 10^16^ cycle requirement for DRAM technology. Compared to solid-solution HZO films, the variety of nano-laminate architectures allowed for a modest increase in endurance. The perspective of free energy based on Landau theory was also reported for explaining ferroelectrics and paraelectrics. The energy curve can be flattened by stacking a ferroelectric with two stable potential wells and a paraelectric with the lowest energy at the zero-polarization value, as shown in Fig. 6c. Alternatively, the mixed phase (ferroelectric + paraelectric) exhibits an intermediate free energy state between the ferroelectric and paraelectric energy states. Additionally, it can be seen in the C-V curve that the HZO-ZrO_2_ laminate thin film's maximal permittivity converges at roughly 0 V (as shown in Fig. 6d). The capacitance of the HZO-ZrO_2_ super-lattices is higher than those of ferroelectric HZO and ferroelectric ZrO_2_ films with the same physical thickness [16]. As a result, as seen in Fig. 6, the capacitance dropped linearly with more lamination (e). As the thickness decreases, the dielectric constant rises in traditional dielectrics, which are the opposite. The impact of surface energy on thickness can account for this. The influence of surface energy is particularly strong in ultrathin films. The highly symmetric cubic phase is stabilized, resulting in paraelectricity, as the thickness of the thin film of the perovskite structure diminishes, whereas the o-phase is stabilized in the fluorite structure. HZO is a strong contender for applications requiring ultrathin ferroelectric materials as a result.

#### Effect of dopants on achieving high permittivity in hafnia ferroelectrics

The doping of perovskite ferroelectrics has been focused on enhancing the piezoelectric constant by considering the electronegativity and elastic modulus of the dopant. However, in the HZO system, Hf and Zr have very similar physical and chemical properties; therefore, different approaches are required. The grain boundary energy and surface energy with doping concentration are the sources of the well-known MPB behavior in the HZO system. This explains why, in contrast to perovskite materials, fluorite materials have a relatively large dependence on thickness whereas the dielectric constant increases with decreasing thickness. The effects of various dopants (Si, Al, Y, Gd, La, Sr) and defects (oxygen vacancies, carbon, hydrogen, and nitrogen) on ferroelectric thin films were systematically analyzed [52, 53]. This can also be utilized to create HZO's anti-ferroelectric properties, which are employed to create an MPB thin film, the boundary between ferroelectric and anti-ferroelectric properties. O-phase stabilization is facilitated by the use of dopants with high atomic radii and low electronegativity. This is brought on by the length of the metal–oxygen connection and dopant dispersion in space. The doping level must be both high enough to overcome the amorphous phase and shift to the m-phase after heat treatment and low enough to shift to the o-phase for this effect to be present, but it may be diminished with a greater atomic radius.

**Fig. 7:**
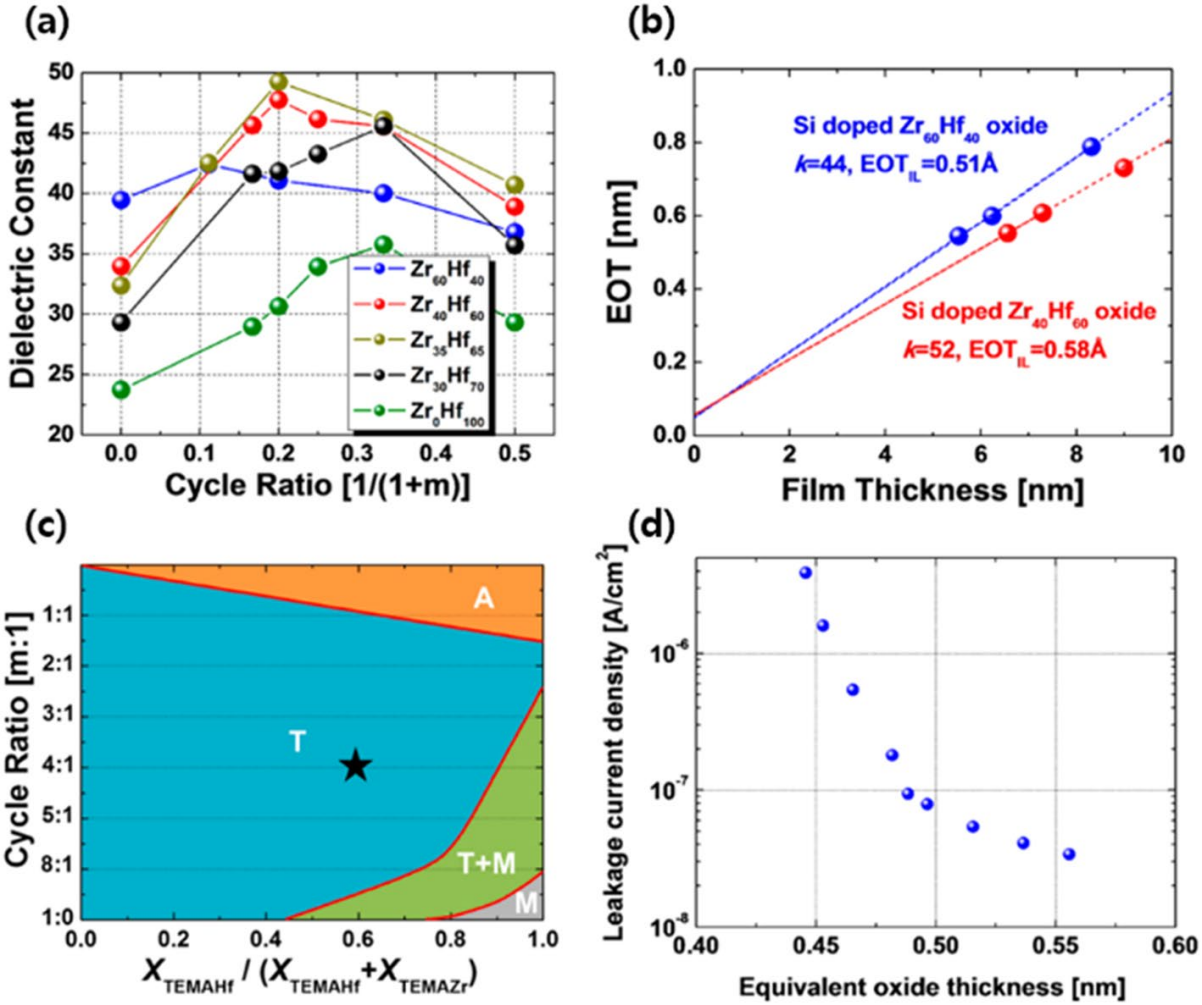
Dopant dependence of HZO. **a** Dielectric constant of the Si-doped Zr_1−x_Hf_x_O_2_ thin films with ALD deposition cycle ratio [Si /(Si + ZrHf cycles)] and **b** EOT values with thin film thickness. **c** Phase diagram of the Si-doped HZO with different Hf fraction and **d** leakage current density of Si-HZO with EOT (measured at 0.8 V). Reprinted with permission from Ref. [53]. Copyright 2015 American Chemical Society

Si-doped HZO thin films of various compositions were investigated to enhance the dielectric properties of ferroelectric capacitors with CMOS-compatible TiN electrodes. Under the optimum conditions of the Si dopant, the Hf-rich HZO films demonstrated a high dielectric constant of 52. As the amount of Si dopant increased, the dielectric constant increased because of the enhanced tetragonal phase fraction. Further increase in the Si dopant concentration led to a decrease in the dielectric constant, as shown in Fig. 7a. The effects of Si doping in Zr-rich and Hf-rich HZO films were compared, and it was found that the Hf-rich HZO films showed a high dielectric constant [52] when compared with the dielectric constant [44] of Zr-rich HZO films [54]. However, interestingly, the EOT was found to be lower in the case of the Zr-rich HZO films than in the Hf-rich HZO films, as shown in Fig. 7b. The crystallinity versus cycle ratio mapping is shown in Fig. 7c. As the doping of Si increased in the HZO films, the phase formation (crystallinity) changed from monoclinic to tetragonal. Further increasing the doping of Si leads to the degradation of the crystal structure, resulting in the formation of an amorphous phase. Therefore, the variation of dielectric constant is based on the phase of the Si-doped HZO films. Hence, the dielectric constant increases during the phase transformation from monoclinic to tetragonal and then decreases when the structure becomes amorphous. Figure 7d presents the EOT versus the leakage current density for various Si-doped HZO films. The lowest EOT (< 0.5 nm) was obtained at a low leakage current density (< 10^−7^ A/cm^2^) with a CMOS-compatible TiN electrode. Hence, Si-doped HZO films are promising candidates for future applications in DRAM technology.

#### The Influence of composition on fluorite structure hafnia to achieve MPB

**Fig. 8:**
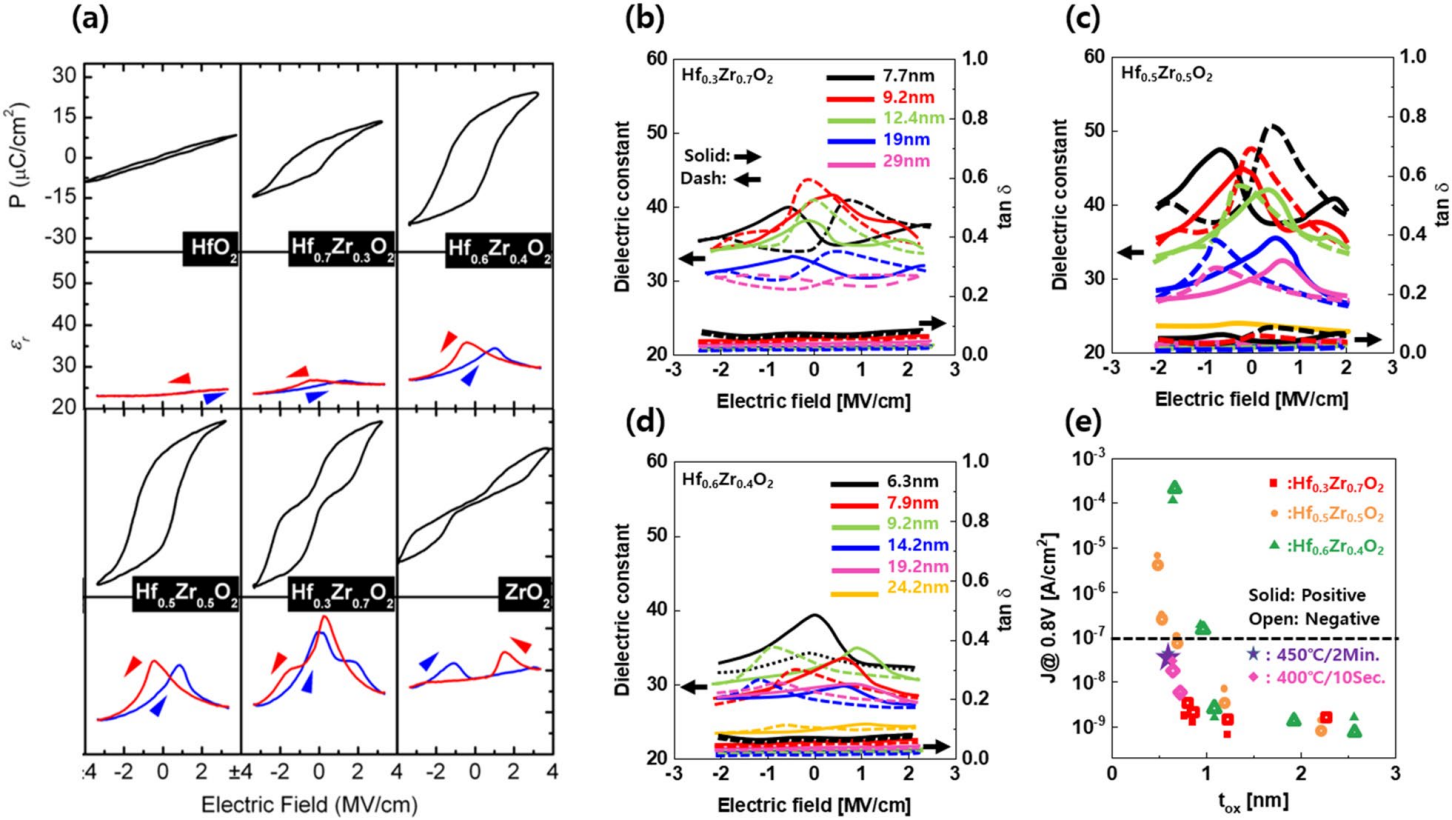
Electrical characteristics of 9 nm thick HZO with various composition ratio. Electrical characteristics of 9 nm-thick HZO with various composition ratio. PE and CV curves were measured at 1 and 10 kHz respectively. Reprinted with permission from Ref. [54]. Copyright [2012] American Chemical Society

According to previous studies, the MPB of the HZO system occurs at a relatively high Zr ratio. It is known that the higher the Zr ratio and the lower the thin film thickness are, the higher is the t-phase. The experimental results show that the MPB has a Zr-rich ratio, similar to that found in previous studies. The P–E and C–E curves revealed the formation of various phases in the hafnia material system with increasing Zr content, as shown in Fig. 8. Depending on the Zr content, the electrical characteristics changed from paraelectric HfO_2_ to ferroelectric HZO to the anti-ferroelectric-like behavior of ZrO_2_. Pure HfO_2_ exhibits paraelectric behavior, thus showing no hysteresis curve in both P–E and C–E curves, even after the applying of an electric field. Ferroelectric characteristics, including an ideal P–E hysteresis curve and a C–E butterfly curve, were formed when the Zr concentration was increased by 50%. Furthermore, compared to ferroelectric and anti-ferroelectric films, the MPB, which is formed when the Zr content is increased by more than 75%, has a very high capacitance at zero electric field. The absence of Hf in pure ZrO_2_ films, however, results in anti-ferroelectric behavior, which means that even after an applied electric field, there is zero remanent polarization [55]. In a hafnia material system, between the FE o-phase and the t-phase, Park et al. reported using the MPB. In perovskite materials, the value dropped with decreasing film thickness, whereas the value increased with decreasing film thickness in the range of 5–20 nm. The phase transition from ferroelectric o-phase to anti-ferroelectric t-phase with a reduction in film thickness from 10 to 5 nm is the most important discovery. Depending on the film thickness, the MPB phase development can be seen in each HZO film composition. The increased κ (~ 50) value and minimum EOT (0.62 nm near the MPB) obtained in this work could be useful in high-capacitance dielectric capacitors, especially in DRAM technology [12]. In conclusion, the HZO composition, film thickness, and annealing temperature affect the phase evolution and κ value of the capacitors with an increase in the MPB.

#### Role of deposition temperature engineering to achieve MPB in Al doped hafnia ferroelectrics

**Fig. 9:**
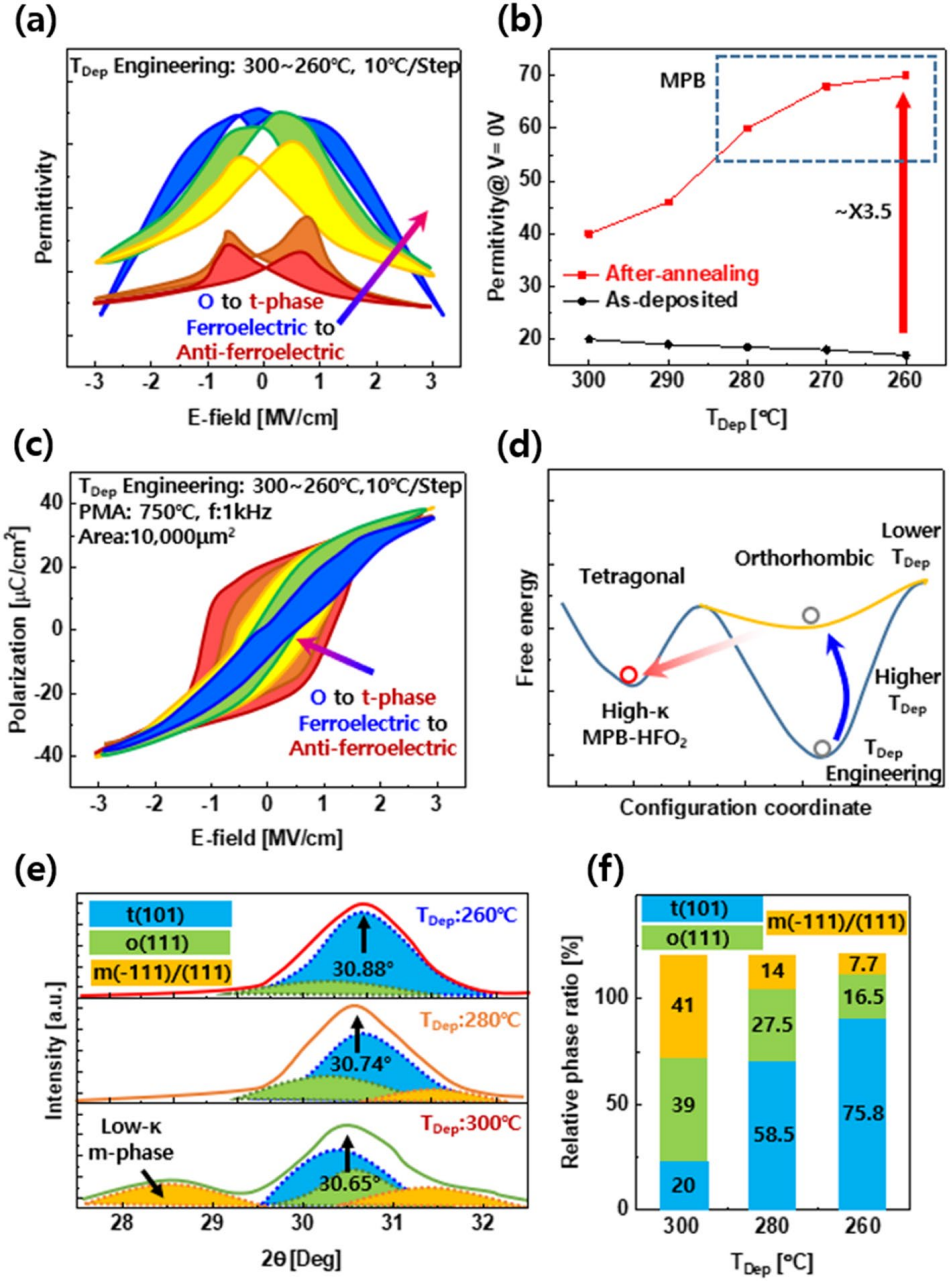
Deposition temperature engineering on Al-HfO_2_. **a** Free energy landscape with deposition temperature. **b** Permittivity-electric field curves and **c** extracted value at 0 V with deposition temperature. **d** and **e** show PE curves and extracted switching probability. **f** GIXRD and **g** the derived relative phase fraction of HAO film. Redrawn from Ref. [19]

As the ALD process is based on the thermal decomposition of C-containing precursors and their reaction with plasma radicals, the amount of defects was analyzed with respect to the process temperature. These defects lead to the increase in the leakage current of the thin film but inhibit crystal growth and promote the formation of a preceding t-phase that can be converted into an o-phase during the heat treatment process. Therefore, the deposition temperature should be considered for MPB formation. In support of this, Zhou et al. reported Al-doped HfO_2_ capacitors with the highest permittivity (~ 68) near the MPB using deposition temperature engineering. The dielectric constant (~ 68) obtained in this study was close to the theoretical limit of HfO_2_ [19, 69]. In Fig. 9a, the t-phase should be larger than the o-phase to form the MPB. This can be used to reduce the free energy of the t-phase via deposition temperature engineering. Figure 9b and c shows the C–V curve and the extracted dielectric constant values while decreasing the deposition temperature from 300 to 260 °C with a 10 °C step, respectively. It is clear that the capacitance increases as the deposition temperature decreases, as well as the phase transformation from ferroelectric to anti-ferroelectric t-phase. As the deposition temperature decreased, the permittivity increased drastically, as shown in Fig. 9b. The effect of the deposition temperature can be clearly observed in the obtained permittivity values, which are 3.5 times higher than those of the as-deposited samples. Similar to the results of other studies, a decreased P_r_ and the typical pinched polarization curve can be observed in Fig. 9d as it approaches the MPB, and the double-switching characteristic is also prominent in the switching probability analysis (Fig. 9e). Furthermore, grazing-induced X-ray diffraction was employed in Fig. 9f to extract material properties from the sample, and the retrieved phase fraction data (shown in Fig. 9g) demonstrates the rise in the t-phase as the deposition temperature dropped. This has to do with the thermal degradation of the precursor, which is a raw material for the ALD process and contains carbon (C). Remaining C, which functions as a defect, can restrict crystal development. For upcoming ultra-low-power transistors and ultra-high-density memory, the development of Al-doped HfO_2_ capacitors with the maximum permittivity [67] is a promising candidate.

#### Influence of ozone dose/ pulse time on the permittivity of hafnia ferroelectrics

**Fig. 10:**
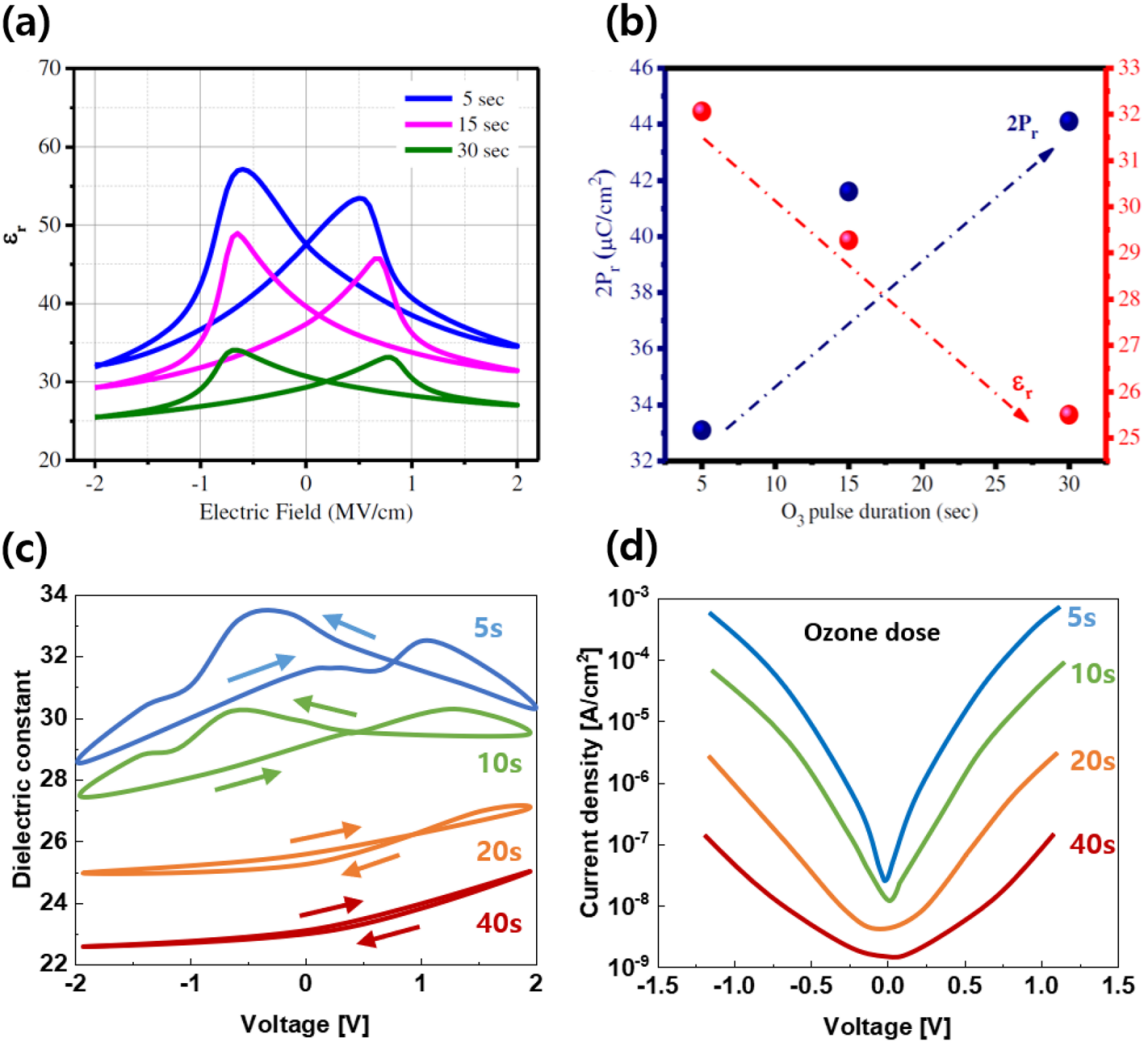
The influence of ozone dose/pulse time on the permittivity of hafnia ferroelectric during the deposition process. **a** Dielectric constant and **b** 2P_r_ of HZO (Hf;Zr = 1:1) with different ozone pulse time. **c** к-V and **d** current density with ozone dose on HfO_2_ thin film. **a** and **b** were reprinted with permission from Ref. [56]. Copyright 2020 Wiley‐VCH GmbH. **c** and **d** were reproduced from Ref. [57]

Another major defect in fluorite structural ferroelectrics is the presence of oxygen vacancies (V_o_). In general, V_o_ in ZnO semiconductor materials is the main reason for the leakage current path; thus, hydrogen atmosphere high-pressure heat treatment has been proposed to reduce it [58, 59]. However, Vo can prevent undesirable phases from forming in ferroelectrics and promote the orthorhombic phase. Therefore, we need to consider the appropriate supply of V_o_ in hafnia. In line with this approach, previous studies have analyzed the behavior in thin film with the amount of ozone dose during the ALD process [60]. The increased ozone pulse period promotes the phase transition from the t- to o- and m-phases by reducing V_o_ in the HZO system, as shown in Fig. 10a, and being extracted, as shown in Fig. 10b. It is revealed that the P_r_ characteristic, which is a ferroelectric index, increases. However, as shown in Fig. 10a and b, the dielectric constant decreases as the ozone pulse period increases. As shown in Fig. 10c and d, when ozone concentration increases, the dielectric constant decreases as the proportion of the t-phase decreases, and a low leakage current owing to the relatively small V_o_ is observed. As a result, ozone had a significant effect on V_o_ in thin film during the deposition. This causes oxygen scavenging at the interface with the electrode during the subsequent heat treatment process or the redistribution of V_o_ under repeated e-field cycles.

#### Role of thermal annealing techniques on achieving MPB in Hafnia ferroelectrics

**Fig. 11:**
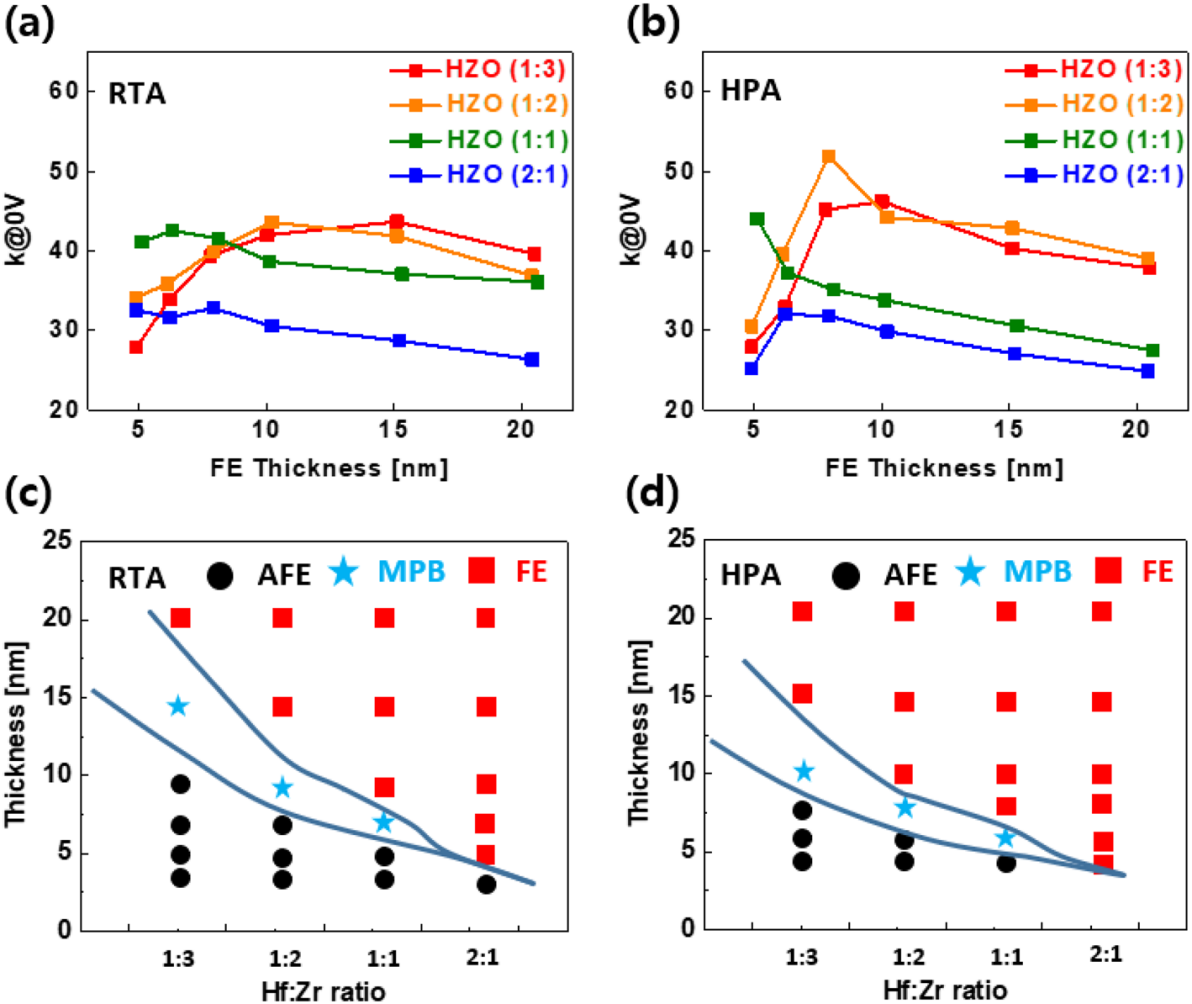
Comparison of dielectric constant and crystal phase between RTA and HPA-treated Hafnia**,** Dielectric constant versus electric field (κ–E) curves for different HZO compositions and thickness with **a** RTA and **b** HPA. **c** and **d** show the phase transition tendency with thickness and composition of HZO system for RTA and HPA respectively. **a** and **b** were reproduced from Ref. [60]

In general, ferroelectric hafnia films are significantly influenced by the crystallization process through various annealing techniques [48, 61–63]. In a previous study, the dielectric constants of various HZO systems were investigated using a standard RTA process at 600 °C for 10 s, as exhibited in Fig. 11a. Figure 11b also shows high-pressure annealing (HPA) at 450 °C and 200 atm for 30 min. The HPA technique is known to be effective for the defect treatment and stress engineering of semiconductor thin films. Figure 11c and d show the MPB regions with various process variables (composition and thickness) using RTA and HPA, respectively. HPA-treated hafnia MPB shows significant improvement in the electrical properties of the thin film at a relatively low temperature compared to those obtained with RTA. It was found that the dielectric constant decreases with increasing the physical thickness for the samples of HZO and Hf-rich HZO, whereas the dielectric constant increases with physical thickness for Zr-rich HZO films. The highest dielectric constant was obtained for the HZO (1:2) composition when compared with the other HZO compositions. A systematic process parameter optimization and annealing technique resulted in the formation of MPB. A high dielectric constant [52] was obtained at a physical thickness of approximately 6 nm using the HPA technique [18, 61]. As can be observed in Fig. 11c and d, the MPB formation can be obtained at a lower annealing temperature (450 °C) in the case of HPA when compared to the RTA (600 °C) process. Moreover, the HPA-treated hafnia MPB was obtained at a lower physical thickness than the RTA sample. HPA-treated hafnia shows high o/t-phase fraction and low m-phase portion. In addition, HPA process allows to form the MPB at relatively thin physical thickness, leading to a high dielectric constant and, hence, a low EOT. Therefore, the HPA technique is a key process in obtaining enhanced electrical properties in hafnia compared with the standard RTA technique.

### Possible applications of MPB in hafnia material system

The MPB can meet the needs of a number of industrial applications because to its exceptional electrical qualities. First, it can be utilized for the DRAM cell capacitance in memory applications. From typical SiO_2_, SiN, and Al_2_O_3_ to high-performance materials like ZrO_2_, HfO_2_, and TiO_2_, dielectric oxide thin films have been used as capacitance layers. In addition, a new structure with a composite of the same materials to suppress unexpected crystal growth and various electrodes (TiN, TaN, W, Ru) for stress engineering were adopted. If the physical thickness of the thin film is decreased while keeping the high dielectric constant of the MPB, a new technological breakthrough in the technical constraints of DRAM can be proposed. Nevertheless, a capacitor cell must achieve ultralow J_g_ (10^–7^ A/cm^2^ at the operating voltage of 0.8 V), a low EOT (0.5 nm) at a low physical thickness of 5 nm, and a certain level of capacitance (10 fF/cell) in order to operate at the level required by DRAM to achieve sufficient signal margin against parasitic circuit capacitances and thermal noise. High density (> tens of gigabits), high speed (20 ns), high endurance (> 10^16^ cycles), and economical fabrication should also be considered while evaluating reliability.

**Fig. 12:**
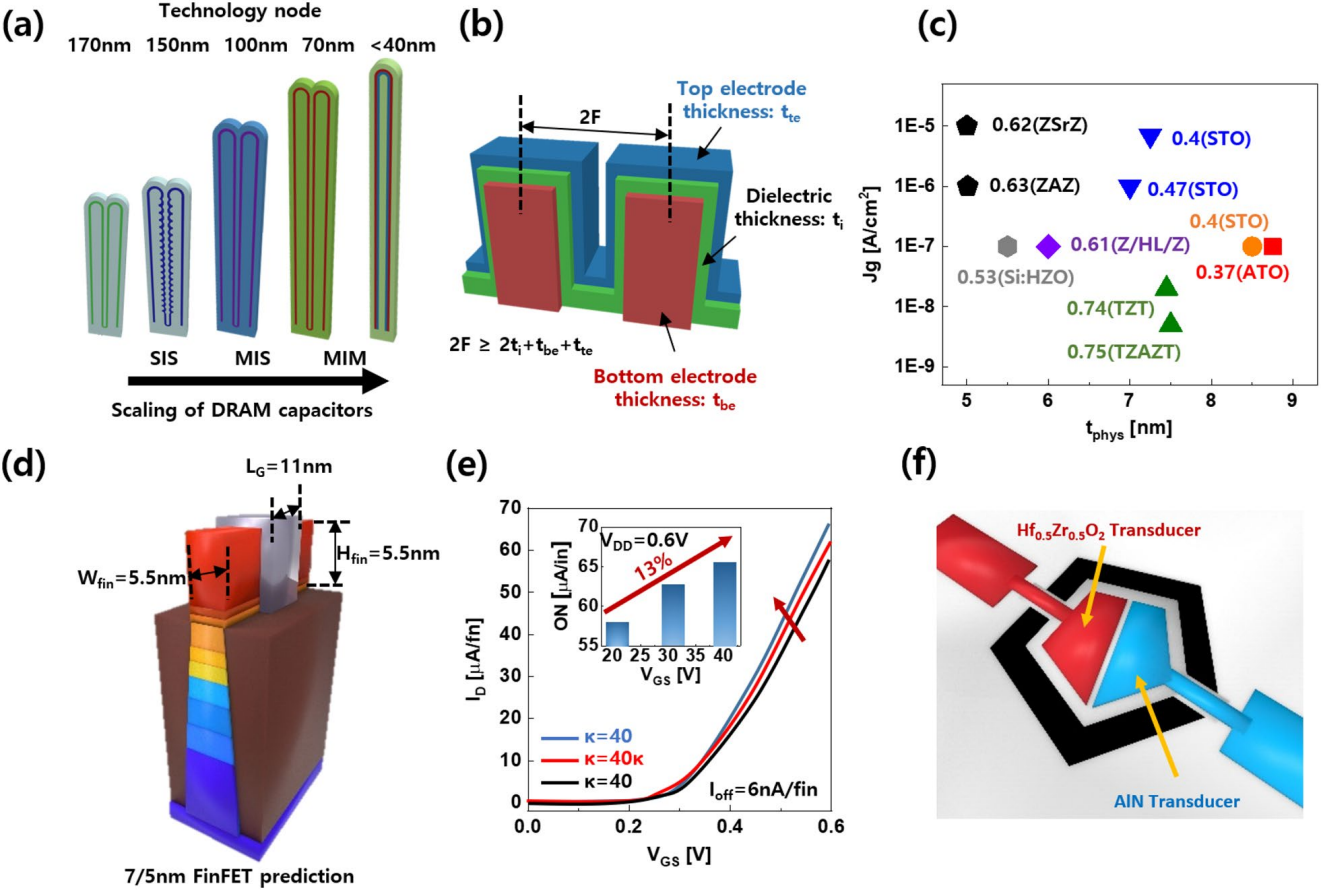
Applications of high-performance hafnia ferroelectrics. Schematic of **a** technology node on DRAM (dynamic random-access memory) capacitor (SIS: silicon–insulator–silicon MIS: metal–insulator–silicon, MIM: metal–insulator–metal) and **b** pillar-structured capacitor (F: minimum feature size). **c** Gate leakage current density versus physical thickness with various material and structures (Z: ZrO_2_, S: Sr, T: TiO_2_, H: HfO_2_ A: Al_2_O_3_, ATO: Al doped TiO_2_). **d** Device structure of FinFET and **e** TCAD simulation result for FinFET. **f** Schematic of HZO-AlN ultra sonic transducer. Figure 12 were redrawn from Ref. [13, 43, 64]

Cell capacitors with a variety of architectures (SIS: silicon-insulator-silicon; MIS: metal–insulator-silicon; MIM: metal–insulator-metal) have been introduced to address these challenges despite the necessity for new materials (Fig. 12a). As seen in Fig. 12, HZO is still making an appeal for compatibility with traditional semiconductor processes and design rule compliance Fig. 12b. It is easily manufactured using ALD and possesses non-volatile characteristics as well as a high dielectric constant for DRAM cells. For comparison with other materials and their composites, as illustrated in Fig. 12, the unresolved problem of the leakage current characteristics of HZO should be resolved Fig. 12c. The current FinFET structure and the TCAD simulation results are shown in Fig. 12d and e. Through the ALD process, the HZO MPB can be used to create a variety of structures, including MFIS and MFFM. Advanced FinFETs with high-κ exhibit strong electrostatic properties and significant driving current [27, 65]. The driving current in the ON state increased by 13% when was raised from 21 to 40. SS fell from 71 to 77 mV/dec and DIBL decreased from 73 to 62 mV/V. These results suggest that a novel approach for the HKMG transistors of the future is to fabricate high-transistor transistors with the MPB.

Finally, the fluorite-structured ferroelectric could be used in flexible devices and large-area integration procedures because, in contrast to commercially available bulk PZT, it can preserve ferroelectric characteristics even in a thin film. The ultrasonic sensor (Fig. 10f) was made out of a 10 nm thick HZO film [47]. The MPB region also has the highest values of the piezoelectric constant d_33_, a measure of piezoelectric performance. So even in the ultrasonic and infrared spectrums, the sensor utilizing the MPB can be used with high sensitivity piezoelectric pressure and temperature sensors. It can therefore be used to create user-friendly interfaces, energy harvesters, electronic skins for robots, healthcare applications, and next-generation display technology that mimics nature’s tactile perception [66–70].

### Critical issues (scaling, leakage, reliability) of DRAM technology

For DRAM technology, a high dielectric constant value has been attained by optimizing a variety of high-performance materials and associated processing factors. There aren’t many methods, though, that work with standard semiconductor techniques. In this view, HZO thin films are still desirable choices for thin films smaller than 10 nm due to their structural robustness and minimal reactivity at the electrode interface (mostly TiN). However, some problems need to be solved before the MPB can take the place of standard high-thin films. Another factor in the failure to reach the required cell capacitance (10 fF/cell) is the DRAM cell capacitor's reduced size. A decrease in the physical thickness of the cell capacitors also results in an increase in the leakage current density. The work function of the electrode and interface between the dielectric should be high in order to achieve the current density requirement in DRAM technology (10^–7^ A/cm^2^ at 0.8 V). Despite the ferroelectric ultrathin film's high dielectric constant characteristic having been described [13, 25, 71], it must also meet the market's endurance standards. Moreover, the reliability characteristics of the DRAM cell capacitor should be met, and in particular the ultimate endurance should be 10^16^ cycles. However, recent reports on hafnia material systems indicate that the maximum endurance is approximately 10^11^ cycles. In addition, a sufficient data retention time has become more challenging. As a result, selecting a high-quality material, optimizing various dielectric material process parameters, and selecting a suitable electrode material are critical for DRAM cell capacitors.

## Summary and outlook

The discovery of ferroelectric properties in the hafnia material system has enabled advancements in memory technology. The hafnia material system’s MPB phase allows for an increase in the dielectric constant, creating new opportunities for DRAM technology. Fluorite-structured ferroelectrics outperform perovskite ferroelectrics in this regard. In contrast to perovskite ferroelectrics, the dielectric constant rises in ferroelectrics with fluorite structures as the film thickness decreases, though the leakage current density rises as the dielectric layer becomes thinner. So the right material, capacitor structure, electrodes, and process parameters must be tuned in order to address the leakage current issue. Additionally, 3D structured capacitors are employed to achieve the required capacitance for high-density DRAM cells in a constrained space. A further rise in the aspect ratio is challenging, nevertheless, due to the storage node's structural fragility. Therefore, the adoption of a capacitor structure with a high aspect ratio and the high density of DRAM cells from 8F^2^ to 6F^2^ in the 1_X_ nm (20 nm) class 3D transistor is required for commercialization. Cell capacitors’ usage of ultrathin insulators causes a quick rise in leakage current, a decline in retention properties, and a reduction in sensing margin. The 1 T-1C structure of the current DRAM needs to have non-volatile functionality added to the insulating film. In particular, it should minimize refresh operations and exhibit non-volatile properties to enable high-performance, ultra-power-efficient DRAM devices for the IoT age. There are currently a few major hurdles to scaling 1 T-1C DRAM technology. Leakage current in small cell size of silicon-based transistors is the primary issue, followed by the large area consumption of storage capacitors. To overcome the limitations of traditional 1 T-1C DRAM technology, IGZO-based capacitor-less DRAM cell (2 T-0C) has recently been proposed. Since the parasitic capacitance of the read transistor act as the storage element, it doesn’t require additional storage capacitor. The 2 T-0C further offers low off-current, which improves retention of the memory cell. Considering these advancements in DRAM technology, stack of an individual DRAM cells leads to high density 3D DRAM. While we continue to create novel high-density DRAM technologies, scaling issues for moving to 1 × nm beyond DRAM remain a major concern. Since the process innovation does not seem to be sufficient to prolong DRAM, high density memory will inevitably require technology open innovations in all areas of process, device, and design. Such high-density DRAM technology may be useful for various applications such as artificial intelligent (AI), intern-of-Things (IoT), cloud/edge computing, and 5G technology. Here, the ferroelectric Hafnium oxide-based functional dielectrics are ideally suited for next-generation DRAM-oriented dielectrics due to their good CMOS process compatibility, non-volatile properties, excellent durability, and quick switching speed. Furthermore, piezoelectric devices (sensors and actuators), nanoscale transistor technology, and intelligent systems can all benefit from the high dielectric constant of such hafnia material systems. FinFET technology, which is very beneficial for nanoscale transistors in semiconductor technology, may also leverage the MPB phenomena.

## Data Availability

Not applicable.

## References

[1] Mitsui T, Warlimont H, Martienssen W (2018). Ferroelectrics and antiferroelectrics. Springer handbook of materials data.

[2] Mikolajick T (2021). Next generation ferroelectric materials for semiconductor process integration and their applications. J. Appl. Phys..

[3] Gao J (2011). Microstructure basis for strong piezoelectricity in Pb-free(Zr0. 2Ti0. 8) O3-(Ba0. 7Ca0. 3) TiO3 ceramics. Appl. Phys Lett..

[4] Ahart M (2008). Origin of morphotropic phase boundaries in ferroelectrics. Nature.

[5] Iwata M, Ishibashi Y (2000). Theory of morphotropic phase boundary in solid solution systems of perovskite-type oxide ferroelectrics: engineered domain configurations. Jpn. J. Appl. Phys..

[6] Chung CC. Microstructural evolution in lead zirconate titanate (PZT) piezoelectric ceramics (2014)

[7] Böscke T (2011). Ferroelectricity in hafnium oxide thin films. Appl. Phys. Lett..

[8] Gaddam V, Das D, Jeon S (2020). Insertion of HfO 2 seed/dielectric layer to the ferroelectric HZO films for heightened remanent polarization in MFM capacitors. IEEE Trans. Electron Devices.

[9] Das D, Gaddam V, Jeon S (2019). Demonstration of high ferroelectricity (P $ _ {r} $~ 29$\mu $ C/cm 2) in Zr Rich Hf x Zr 1–x O 2 Films. IEEE Electron Device Lett..

[10] Park MH (2015). Ferroelectricity and antiferroelectricity of doped thin HfO2-based films. Adv. Mater..

[11] Park MH (2017). A comprehensive study on the structural evolution of HfO 2 thin films doped with various dopants. J. Mater. Chem. C.

[12] Park MH (2018). Morphotropic phase boundary of Hf1–x Zr x O2 thin films for dynamic random access memories. ACS Appl. Mater. Interfaces..

[13] Kim SK, Popovici M (2018). Future of dynamic random-access memory as main memory. MRS Bull..

[14] Kashir A, Farahani MG, Hwang H (2021). Towards an ideal high-κ HfO 2–ZrO 2-based dielectric. Nanoscale.

[15] Kashir A, Hwang H (2021). A CMOS-compatible morphotropic phase boundary. Nanotechnology.

[16] Cheema SS (2022). Ultrathin ferroic HfO2–ZrO2 superlattice gate stack for advanced transistors. Nature.

[17] Kim S (2021). Method to achieve the morphotropic phase boundary in Hf x Zr 1–x O 2 by electric field cycling for DRAM cell capacitor applications. IEEE Electron Device Lett..

[18] Das D (2021). Sub 5 Å-EOT Hf_x_Zr 1–x O_2_ for next-generation DRAM capacitors using morphotropic phase boundary and high-pressure (200 atm) annealing with rapid cooling process. IEEE Trans. Electron Devices.

[19] Zhou J, Zhou J (2021). Al-doped and deposition temperature-engineered HfO 2 near morphotropic phase boundary with record dielectric permittivity (~ 68). 2021 IEEE International Electron Devices Meeting (IEDM).

[20] Yim K (2015). Novel high-κ dielectrics for next-generation electronic devices screened by automated ab initio calculations. NPG Asia Mater..

[21] Choi J, Mao Y, Chang J (2011). Development of hafnium based high-k materials—a review. Mater. Sci. Eng. R. Rep..

[22] Lee SW (2011). Atomic layer deposition of SrTiO3 thin films with highly enhanced growth rate for ultrahigh density capacitors. Chem. Mater..

[23] Kim SK (2008). Al-doped TiO2 films with ultralow leakage currents for next generation DRAM capacitors. Adv. Mater..

[24] Jeon W (2014). Evaluating the top electrode material for achieving an equivalent oxide thickness smaller than 0.4 nm from an Al-doped TiO2 film. ACS Appl. Mater. Interfaces..

[25] Popovici M, Popovici M (2018). High-performance ($\text {EOT}< 0.4\text {nm} $, Jg∼ 10− 7 A/cm 2) ALD-deposited Ru\SrTiO 3 stack for next generations DRAM pillar capacitor for next generations DRAM pillar capacitor. 2018 IEEE International electron devices meeting IEDM.

[26] Goh Y (2020). Crystalline phase-controlled high-quality Hafnia ferroelectric with RuO_2_ electrode. IEEE Trans. Electron Devices.

[27] Kittl J (2009). High-k dielectrics for future generation memory devices. Microelectron. Eng..

[28] Shirane G, Suzuki K, Takeda A (1952). Phase transitions in solid solutions of PbZrO3 and PbTiO3 (II) X-ray study. J. Phys. Soc. Jpn..

[29] Sawaguchi E (1953). Ferroelectricity versus antiferroelectricity in the solid solutions of PbZrO3 and PbTiO3. J. Phys. Soc. Jpn..

[30] Jaffe B, Cook W, Jaffe H, Jaffe B (1971). The piezoelectric effect in ceramics. Piezoelectric ceramics.

[31] Ishibashi Y, Iwata M (1998). Morphotropic phase boundary in solid solution systems of perovskite-type oxide ferroelectrics. Jpn. J. Appl. Phys..

[32] Singh DJ (2006). Role of A-site and B-site ions in perovskite ferroelectricity. Ferroelectrics..

[33] Park MH (2018). Review and perspective on ferroelectric HfO 2-based thin films for memory applications. MRS Commun..

[34] Gaddam V (2021). Ferroelectricity enhancement in Hf 0.5 Zr 0.5 O 2 based tri-layer capacitors at low-temperature (350 C) annealing process. IEEE Electron Device Lett..

[35] Das D, Gaddam V, Jeon S (2021). Ferroelectricity in Al2O3/Hf0. 5Zr0. 5O2 bilayer stack: role of dielectric layer thickness and annealing temperature. J. Semicond. Technol. Sci..

[36] Das D (2020). Trade-off between interfacial charge and negative capacitance effects in the Hf-Zr-Al-O/Hf0. 5Zr0. 5O2 bilayer system. Solid State Electron..

[37] Buyantogtokh B, Gaddam V, Jeon S (2021). Effect of high pressure anneal on switching dynamics of ferroelectric hafnium zirconium oxide capacitors. J. Appl. Phys..

[38] Das D, Gaddam V, Jeon S (2021). Insertion of dielectric interlayer: a new approach to enhance energy storage in Hf_x_Zr 1–x O_2_ capacitors. IEEE Electron Device Lett..

[39] Das D (2021). Influence of high-pressure annealing conditions on ferroelectric and interfacial properties of Zr-Rich Hf_x_Zr_1-X_ O_2_ capacitors. IEEE Trans. Electron Devices.

[40] Jeon S, Das D, Gaddam V, Jeon S (2020). Effect of high pressure annealing temperature on the ferroelectric properties of TiN/Hf 0.25 Zr 0.75 O 2/TiN capacitors. 2020 4th IEEE Electron Devices Technology & Manufacturing Conference (EDTM).

[41] Henriques A (2014). Crystallographic changes in lead zirconate titanate due to neutron irradiation. AIP Adv..

[42] Materlik R, Künneth C, Kersch A (2015). The origin of ferroelectricity in Hf1− xZrxO2: A computational investigation and a surface energy model. J. Appl. Phys..

[43] Aksel E, Jones JL (2010). Advances in lead-free piezoelectric materials for sensors and actuators. Sensors.

[44] Ni K, Ni K (2019). Equivalent oxide thickness (EOT) scaling with hafnium zirconium oxide high-κ dielectric near morphotropic phase boundary. 2019 IEEE international electron devices meeting (IEDM).

[45] Park MH (2019). Thermodynamic and kinetic origins of ferroelectricity in fluorite structure oxides. Adv. Electron. Mater..

[46] Ku B, Ku B (2020). Fast thermal quenching on the ferroelectric Al: HfO2 thin film with record polarization density and flash memory application. 2020 IEEE symposium on VLSI technology.

[47] Ku B (2022). Improved ferroelectric characteristics of ALD lanthanum-doped hafnium oxide thin film by controlling post-cooling time. Appl. Surf. Sci..

[48] Hwang J (2022). Relatively low-k ferroelectric nonvolatile memory using fast ramping fast cooling annealing process. IEEE Trans. Electron Devices.

[49] Kim Y (2014). Origins of domain wall pinning in ferroelectric nanocapacitors. Nano Converg..

[50] Weeks SL (2017). Engineering of ferroelectric HfO2–ZrO2 nanolaminates. ACS Appl. Mater. Interfaces..

[51] Park JY (2021). Engineering strategies in emerging fluorite-structured ferroelectrics. ACS Appl. Electron. Mater..

[52] Park MH (2020). Review of defect chemistry in fluorite-structure ferroelectrics for future electronic devices. J. Mater. Chem. C.

[53] Park MH, Hwang CS (2019). Fluorite-structure antiferroelectrics. Rep. Prog. Phys..

[54] Ahn J-H, Kwon S-H (2015). Sub-0.5 nm equivalent oxide thickness scaling for Si-Doped Zr1–x Hf x O2 thin film without using noble metal electrode. ACS Appl. Mater. Interfaces..

[55] Muller J (2012). Ferroelectricity in simple binary ZrO2 and HfO2. Nano Lett..

[56] Kashir A, Oh S, Hwang H (2021). Defect engineering to achieve wake-up free HfO2-based ferroelectrics. Adv. Eng. Mater..

[57] Pal A (2017). Enhancing ferroelectricity in dopant-free hafnium oxide. Appl. Phys. Lett..

[58] Oh S-I (2013). Hydrogenated IGZO thin-film transistors using high-pressure hydrogen annealing. IEEE Trans. Electron Devices.

[59] Kim T-W (2015). Impact of H 2 high-pressure annealing onto InGaAs quantum-well metal–oxide–semiconductor field-effect transistors with Al 2 O 3/HfO 2 gate-stack. IEEE Electron Device Lett..

[60] Yoon S-J (2019). Polarization switching kinetics of the ferroelectric Al-doped HfO2 thin films prepared by atomic layer deposition with different ozone doses. J. Vac. Sci. Technol. B. Nanotechnol. Microelectron. Mater. Process. Meas. Phenom..

[61] Das D, Jeon S (2020). High-k Hf x Zr 1–x O_2_ ferroelectric insulator by utilizing high pressure anneal. IEEE Trans. Electron Devices.

[62] Joh H (2021). Flexible ferroelectric hafnia-based synaptic transistor by focused-microwave annealing. ACS Appl. Mater. Interfaces..

[63] Joh H (2021). Low-temperature growth of ferroelectric Hf0. 5Zr0. 5O2 thin films assisted by deep ultraviolet light irradiation. ACS Appl. Electron. Mater..

[64] Ghatge M (2019). An ultrathin integrated nanoelectromechanical transducer based on hafnium zirconium oxide. Nat. Electron..

[65] Yoon C, Moon S, Shin C (2020). Study of a hysteresis window of FinFET and fully-depleted silicon-on-insulator (FDSOI) MOSFET with ferroelectric capacitor. Nano Converg..

[66] Jo S-H (2022). L-shape triple defects in a phononic crystal for broadband piezoelectric energy harvesting. Nano Converg..

[67] Ahmed A (2021). Additively manufactured nano-mechanical energy harvesting systems: advancements, potential applications, challenges and future perspectives. Nano Converg..

[68] Kim TY, Kim SK, Kim S-W (2018). Application of ferroelectric materials for improving output power of energy harvesters. Nano Converg..

[69] Jung M (2020). Flexible multimodal sensor inspired by human skin based on hair-type flow, temperature, and pressure. Flex. Print. Electron..

[70] Jung M (2019). Amorphous FeZr metal for multi-functional sensor in electronic skin. npj Flex. Electron..

[71] Fröhlich K (2011). Low equivalent oxide thickness TiO2 based capacitors for DRAM Application. ECS Trans..

